# The occurrence of mycotoxins in wheat from western Romania and histopathological impact as effect of feed intake

**DOI:** 10.1186/1752-153X-7-99

**Published:** 2013-06-10

**Authors:** Ersilia Alexa, Cristina Adriana Dehelean, Mariana-Atena Poiana, Isidora Radulov, Anca-Maria Cimpean, Despina-Maria Bordean, Camelia Tulcan, Georgeta Pop

**Affiliations:** 1 Faculty of Food Processing Technology, Banat’s University of Agricultural Sciences and Veterinary Medicine from Timisoara, Calea Aradului no. 119, Timisoara 300645, Romania; 2 Faculty of Pharmacy, Victor Babes University of Medicine and Pharmacy from Timisoara, Eftimie Murgu Square no. 2, Timişoara 300041, Romania; 3 Faculty of Agriculture, Banat’s University of Agricultural Sciences and Veterinary Medicine from Timisoara, Calea Aradului no. 119, Timisoara 300645, Romania; 4 Faculty of Medicine, Victor Babes University of Medicine and Pharmacy from Timisoara, Eftimie Murgu Square no. 2, Timişoara 300041, Romania; 5 Faculty of Veterinary Medicine, Banat’s University of Agricultural Sciences and Veterinary Medicine from Timisoara, Calea Aradului no 119, Timisoara 300645, Romania

**Keywords:** Deoxynivalenol, Zearalenone, Fumonisins, Ochratoxin A

## Abstract

**Background:**

The goal of this study has been to evaluate the extent of mycotoxins contamination and their co-occurrence in wheat grain intended for animal feed. A total of 52 wheat samples were collected from the harvest of two consecutive years (2010, 2011) from two counties (Timis and Arad) located in Western Romania and the presence of ochratoxin A (OTA), deoxynivalenol (DON), zearalenone (ZON) and fumonisins (FUMO) was determined by enzyme-linked immunosorbent assay (ELISA). In order to evaluate the toxicological impact of mycotoxins, naturally contaminated wheat samples were administered in rats feed for one month.

**Results:**

The mycotoxin with the highest incidence in wheat samples was DON due to agro-climatic conditions typical for the west part of Romania. DON was found in 73.08% of samples harvested in 2010 and the highest level of contamination was 3390 ppb. The incidence of DON in sample from 2011 was lower than those of 2010, with a frequency of occurrence of 19.23%. The occurrence of ZON was in the range 69.23–76.92%, with an average value of 187.74 ppb. The OTA content in wheat was below the maximum tolerable limit established by EU Commission regulation for feed legislation (250 ppb). For FUMO the lowest percentage of positive samples was registered (15.38% in wheat sample harvested in 2010 and 11.54% positive samples in 2011). With respect to the co-occurrence of *Fusarium* mycotoxins, the results proved that ZON was found as a co-contaminant together with DON, especially when climatic conditions for development of fungus are favorable (high air humidity). The differences recorded between investigated localities and their classifications according to the mycotoxin kind and year of harvest were carried out by principal components analysis (PCA). The histopathological and immunohistochemical evaluation performed by hematoxylin and eosin (HE) staining technique as well as by assessing the vascular endothelial growth factor (VEGF) revealed significant modification of kidney, liver and spleen cells in the case of DON and FUMO. In terms of toxicity induced by OTA and ZON it was highlighted mixed normal and necrotic areas in liver, while histological assessment revealed normal VEGF expression in kidneys.

**Conclusions:**

Although none of the analyzed samples exceeding the stipulated maximum limits for cereals used as feed, a high incidence of mycotoxins produced by *Fusarium* species have been recorded (DON and ZON) in wheat samples harvested in Western Romania. Also, histopathological evaluation revealed significant tissue lesions in liver and kidney of rats after one month of feeding with natural contaminated wheat.

## Introduction

Cereals and cereal products are significant human food resources and livestock feeds in the whole world. Each year, a large number of crops are susceptible to fungal attack either in the field or during storage, leading to considerable financial losses and damage the health of humans and animals [1]. These fungi may produce, as secondary metabolites a diverse group of chemical substances known as mycotoxins. Mycotoxins are toxic chemical products formed as secondary metabolites by many species of fungi that colonize crops and contaminate them with toxins in the field or after harvest. They are produced during growth and multiplication of fungus when microecological conditions are favorable [2,3]. Practically, there are no known areas in the world without mycotoxins and it is estimated that 25–60% of the world’s grains contaminated with mycotoxins are produced mainly by fungus of the genera *Aspergillus, Fusarium, Penicillium*[4]. The contamination of vegetable products with mycotoxins has been a serious problem in Balkan communities. Several researches on the mycotoxins’ role in endemic kidney disease were geographically limited to the Balkan region [5,6]. Balkan endemic nephropathy (BEN) is found in certain rural areas of the Balkans and affects at least 25 000 inhabitants. A number of descriptive studies have suggested a correlation between the exposure to ochratoxin A (OTA), Balkan endemic nephropathy and the mortality caused by urothelial urinary tract tumors [7-11].

The most important groups of mycotoxins that occur in grain are aflatoxins, ochratoxins, trichotecenes (deoxynivalenol, nivalenol), zearalenone and fumonisins [12].

Ochratoxins are the first major group of mycotoxins identified after the discovery of aflatoxins. Ochratoxin A (OTA) discovered in 1965, is produced on stored cereals by *Penicillium verrucosum* in temperate climates and by several species of *Aspergillus* in products of tropical and subtropical *climates*[13]. OTA acts as a nephrotoxin for all studied animal species but it is also toxic for humans, which have the longest period of elimination from the body [14]. OTA is also a carcinogenic, teratogenic and immunotoxic compound, affecting both humoral and cell-mediated immunity [15].

Another species of fungi responsible for the production of mycotoxins called trichothecenes are *Fusarium sp.*[16,17]. DON is the most frequent trichotecene contaminants of agricultural crops throughout the world and is produced by species such as *Fusarium* g*raminearum* and *Fusarium culmorum.* Extensive survey data indicate the occurrence of this mycotoxin, particularly in wheat and corn [18-22]. DON is a potent antifeedant, inducing in animals, especially in swine, feed refusal and vomiting and can also affect the immune system. In human body, DON causes vomiting, headache, fever and nausea [14,23]*.*

Zearalenone (ZON) is a fungal metabolite, mainly produced by *Fusarium graminearum* and *Fusarium culmorum*, which are known to colonize maize, barley, wheat, oats and sorghum [24]*.* ZON and its related compounds can cause hyperoestrogenism and severe reproductive and infertility problems in animals, especially in swine [25]*.* Regarding the incidence rates and concentration levels in cereals, maize and oats were most frequently contaminated [4,26-29].

Fumonisins (FB1 and FB2) represent a group of mycotoxins characterized at the end of 80s, produced by *Fusarium verticillioides.* Fumonisins are cancer-promoting metabolites of *Fusarium proliferatum* and *Fusarium verticillioides* that have a long-chain hydrocarbon unit which plays a role in their toxicity. Consumption of food contaminated with fumonisins has been associated with elevated human oesophageal cancer incidence. The total intake of FB1 in the European diet has been estimated at 1.4 μg/kg of body weight per week [30].

In Romania, the growing of cereals is one of the main occupations, with economic, social and nutritional relevance. Cereal grain production has increased in recent years due to increased yields. Wheat dominates in the west part of Romania as a primary crop and represents an important part in animal feed [31]. Romania is a major regional producer of wheat, ranking third in Central Europe behind Serbia and Hungary [1].

The presence of mycotoxins in feeds is potentially hazardous to animal’s health. However, there is limited information on the occurrence and co-occurrence of these toxins in cereals that are of prime importance in animal nutrition. The purpose of this study was to assess the extent of mycotoxins contamination and their co-occurrence in wheat grain intended for animal feed and harvested from Western Romania for two consecutive years, 2010 and 2011. Also, it was evaluated the histopathological impact caused by consumption of grains contaminated with mycotoxins.

## Results and discussion

### The occurrence of mycotoxins

The wheat samples were analyzed in order to evaluate the mycotoxins content (ZON, OTA, DON, FUMO). The occurrence of mycotoxins is presented in Table 1 and the co-occurrence in Table 2.

**Table 1:** Frequency of mycotoxins occurrence in wheat grain harvested in Western Romania

**Mycotoxins**	**No. of positive samples (frequency of occurrence %)**	**No of samples over LMA (%)**	**Concentration in samples**	
			Average* (ppb)	**Range (ppb)**
**2010 harvest year**				
OTA	9 (34.62)	–	6.39	3.88–11.3
DON	19 (73.08)	–	2263.1	294–3390
ZON	18 (69.23)	–	187.74	37.65–1000
FUMO	4 (15.38)	–	1102.5	960–1180
**2011 harvest year**				
OTA	24 (92.31)	–	5.71	2.67–25.70
DON	5 (19.23)	–	763.6	254–1440
ZON	20 (76.92)	–	54.54	28.22–105.64
FUMO	–	–	–	–

*Average value of positive samples.

**Table 2:** **The co**-**occurrence of Fusarium mycotoxins in wheat samples**

**Year**	**No. of positive sample DON and ZON (frequency of occurrence %)**	**No. of positive sample DON and FUMO (frequency of occurrence %)**
2010	16 (61.54)	2 (7.69)
2011	2 (7.69)	–

In our study it was determined DON, ZON and FUMO that occur in wheat grain samples as a result of infection of the *living plants* by the relevant Fusarium *species,* and also mycotoxins also occurred during storage (OTA), produced by *Aspergillus* and *Penicillium.*

DON was found in 73.08% of samples harvested in 2010 year, with highest levels of contamination 3.39 ppm. The average value of positive samples was 2.26 ppm. DON is a trichothecene with high incidence in cereals from South East of Europe. Regarding the incidence of DON in wheat sample for food consumption, studies made in Serbia during 2004–2007 highlighted that the incidence rate of this mycotoxin was 50% (the level was in the range 0.63–1.84 ppm), including samples with a contamination level over the maximum admitted level (MAL) established by the European Commission for unprocessed cereals (1.25 ppm) [1,32-35]. Also, in Croatia, for wheat samples for animal feed harvested in 2004, DON levels higher than the limit established by European Commission (8.0 ppm) were recorded [21].

Our study indicates that in 2011 the incidence of DON was lower than in samples harvested in 2010, with a frequency of occurrence of 19.23%. Also, the average of positive samples was lower in 2011 (0.763 ppm) than in 2010 (2.263 ppm). With respect to DON content, the wheat samples are suitable for animal nutrition, none of the samples exceeding the stipulated maximum allowed of 8 ppm for cereals used as feed [35]. The average value of DON in wheat samples harvested in Europe, reported by Binder *et al*., 2007 [18] was 0.37 ppm and the maximum value was 5.51 ppm. In Romania, the level of contamination with DON was higher than that reported in other European countries [26]. Our results were in agreement with those reported in the west part of Romania [36-38]. Previous studies regarding the DON incidence in cereals harvested in Romania, during 2002–2004, reported that the level of positive samples was 56% and the percentage of samples that exceeded the MAL was in the range 25–61% depending on the climate conditions [39].

**Figure 1:**
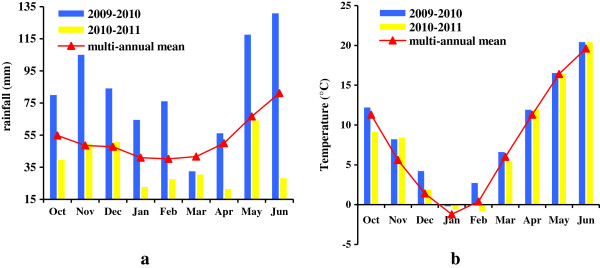
Monthly amounts of rainfall (a) and the mean monthly air temperatures (b) in the west part of Romania relative to multi-annual mean.

Depending on weather conditions, cereals used for feeding may be contaminated above the regulated or recommended limits for mycotoxins. The incidence of mycotoxins in cereals was influenced by seasonal weather conditions. Romania is located in the continental climate characterized by dry and cold winter and hot summer, with optimal conditions for fungal development. The climatic conditions (rainfalls and temperature) registered in the western part of Romania during the investigated period relative to multi-annual mean, as reported by the National Meteorological Administration of Romania [40], are presented in Figure 1(a, b). It can be noticed that temperatures did not record high variations reported to multi-annual mean. The rainfalls (mm) registered in the years 2009 and 2010, during the growing and harvesting of wheat, were higher than the multi-annual mean. In contrast, the values recorded in the same period during 2010–2011 were lower than multi-annual mean. The average temperatures in May-June 2010 were similar with the multi-annual mean, while the amounts of rainfalls were higher than the multi-annual mean. These conditions were favorable for *Fusarium* mycotoxins development, especially DON. In years with rains during blooming and harvesting seasons, the level of cereals contamination with DON can be very high [41]. In 2011, the level of rainfalls, in the same period (May-June) was lower than the multi-annual mean, Figure 1a. Based on these climatic conditions it can be explained our results regarding the high occurrence of DON in 2010 (73.08%) versus 2011 (19.23%).

Considering the importance of agro-climatic and agro-techniques conditions on grain infection with mycotoxins in the field, it is necessary respecting agro-techniques measures as not to create factors favoring the development and growth of fungi, such as: avoid of excessive moisture during vegetation by using of rational irrigation, using of effective fungicides and control of insect attack by cultivation of varieties with genetic resistance to fungal attack. DON frequently co-occurs with another mycotoxin called zearalenone (sex hormone) produced by the same fungus that produces DON [22].

ZON production mainly takes place before harvesting, but may also occurs post harvest if the crop is not handled and dried properly. Based on results presented in Table 1 it can be noticed that ZON was also one of the most common mycotoxins detected in wheat samples. The incidence of ZON in wheat sample was over 69%. In terms of harvest year 2010, ZON was detected in 69.23% of the analyzed samples and the recorded values were in the range 37.65–1000 ppb with an average level of 187.74 ppb. In 2011 the ZON frequency of occurrence was 76.92% and the concentration was in the range 28.22–105.64 ppb. In our study the incidence of ZON was high, but did not exceed the LMA value established by EU regulation for cereals used as animal feed (2 ppm for wheat) [35]. Our results were in agreement with previous reported data regarding the ZON incidence in the Central European area that indicated that nearly half of the investigated samples were contaminated with ZON, the maximum value recorded was 1392 ppb for maize [23]. In Romania, previous studies reported that the contamination level with ZON was usually in the range 10–100 ppb and, for 40% of the investigated wheat samples harvested in 2004, values that exceeded 100 ppb were recorded [39]. Similar results, regarding ZON incidence, were reported by Galbenu *et al*., 2011 [42]*,* when the incidence of ZON was 72.6% for the feed samples and the recorded levels were in the range 1.65–1050 ppb.

Regarding the FUMO content, it can be noticed that the lowest percentage of positive samples was recorded (15.38% in wheat samples harvested in 2010 and 11.54% in 2011). The highest value (1180 ppb) was reached in wheat samples harvested in 2010 from Timis County. Our results are in agreement with previous studies which reported the low incidence of FUMO in Eastern Europe [33,34]. EU regulations not provide MAL in terms of fumonisins level in unprocessed cereals (excluding maize) [35]. The Food and Drug Administration (FDA) has announced guidance levels for total fumonisin levels in corn products in order to protect human and animal health. Thus, the maximum FUMO level recommended by FDA is in the range 2–4 ppm for human food and 5–100 ppm for animal feed depending on the species and the proportion of the contaminated material in the total diets [30].

Since the growing of *Fusarium* mycotoxins is influenced by the field climatic conditions, the higher rainfalls reported in 2010 could be responsible for the fumonisine production.

OTA represents a mycotoxin that accumulates during storage for longer periods when environmental conditions are favorable for molds development. In 2010, OTA incidence in the wheat samples was lower than others mycotoxins (DON and ZON). Thus, the value registered for frequency of occurrence was 34.62% and for average level of contamination was 6.39 ppb. The highest contamination level reached for samples harvested in 2010 was 11.3 ppb. The frequency of occurrence in wheat samples harvested in 2011 was higher than for samples harvested in 2010. Thus, for wheat samples harvested in 2011 was registered 92.31% positive samples and the maximum level recorded for OTA was below 25.70 ppb. Nevertheless, the contamination level in wheat samples were below the maximum tolerable limit of OTA established by EU Commission regulation regarding the animal feed legislation (250 ppb) [35]. The presence of OTA in wheat samples in 2011 and the lower incidence in 2010, when the rainfall was more abundant, indicates that OTA is a mycotoxin that appears mainly during post-harvest storage in unsuitable conditions. In order to avoid the production of mycotoxins after harvest, it is advisable to dry the grains until to optimum moisture (less than 14%). Regarding the OTA level recorded in wheat grains, all analyzed samples have proved to be suitable for animal feed.

In terms of the co-occurrence of *Fusarium* mycotoxins, our results revealed that ZON was found as a co-contaminant together with DON, especially when climatic conditions for development of fungus were favorable (high air humidity). The results were in agreement with previous studies regarding the co-occurence of mycotoxins in wheat samples from Romania [39]*.*

The co–occurrence of mycotoxins can affect both the level of mycotoxins production and the toxicology of the contaminated grains resulting in additive and synergistic effects. Generally, it should be considered that, naturally contaminated feed usually contain more than one mycotoxin. ZON is frequently found as a co-contaminant together with trichothecenes, especially DON [43]. Concerning the co-occurrence of ZON and DON in samples, Table 2, our results indicated that 61.54% of the positive samples harvested in 2010 contain both DON and ZON and only two samples were contaminated by DON and FUMO. For 2011 harvest year, it was observed that 7.69% of the positive samples contain DON and ZON. In this case, no sample was positive for DON and FUMO.

### Principal component analysis (PCA)

**Figure 2:**
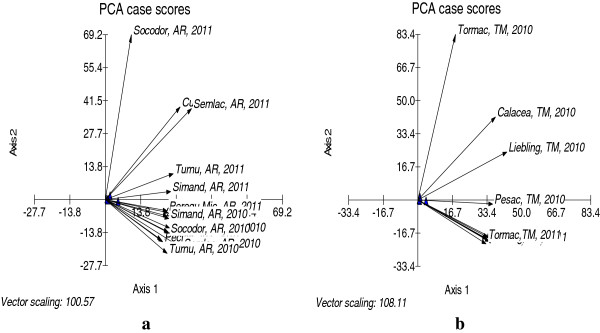
PCA biplot case score representation of the investigated counties (a: Arad County; b: Timis County).

**Figure 3:**
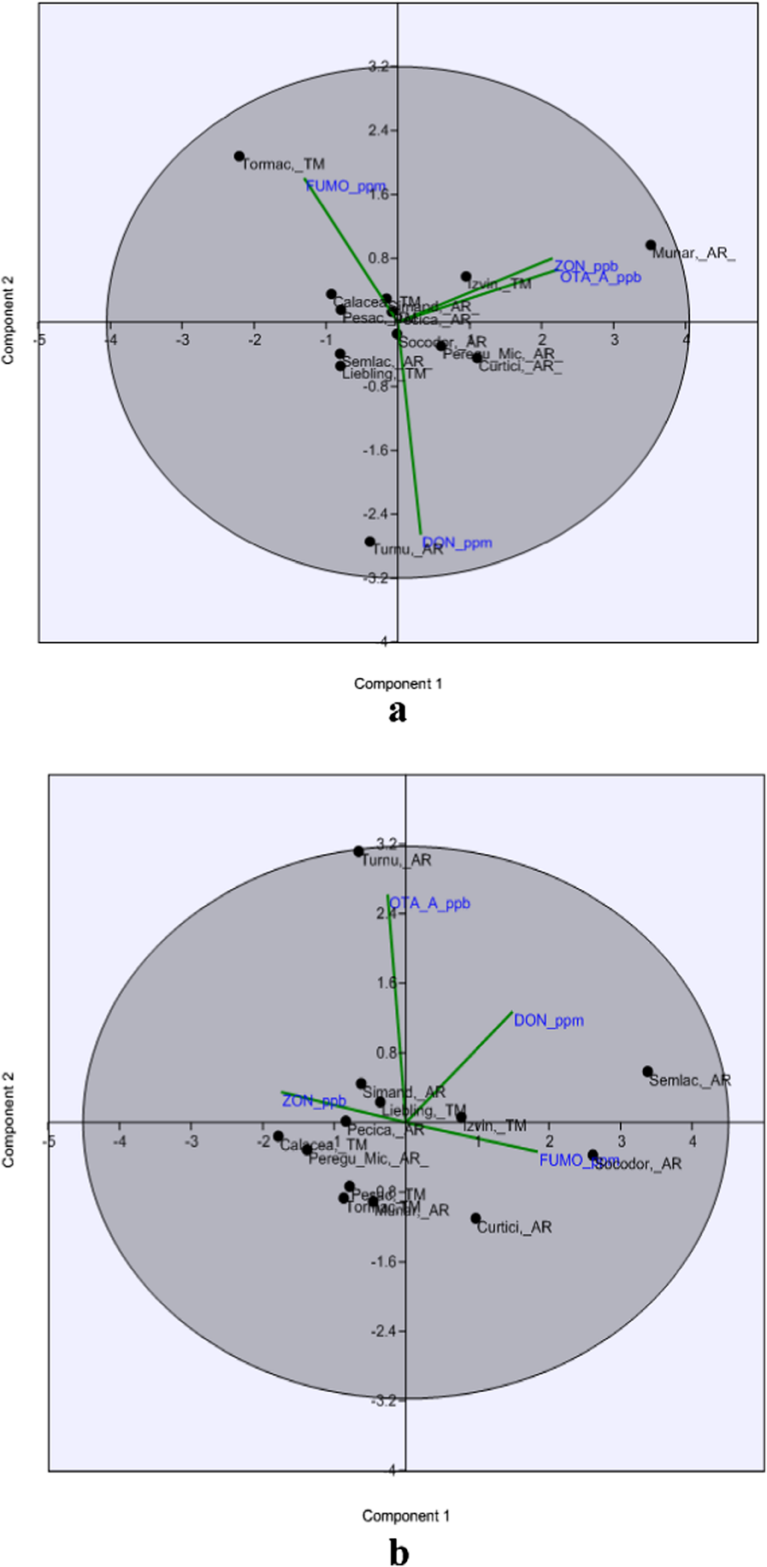
Biplot graphical representation of the Principal Components data corresponding to wheat samples (a: harvested in 2010; b: harvested in 2011).

The differences recorded between investigated localities and their classifications according to the mycotoxin kind and year of harvest were carried out by principal components analysis (PCA). PCA biplot case score representation of the investigated counties was showed in Figure 2 and biplot graphical representation of the Principal Components data corresponding to the years 2010 and 2011 were presented in the Figure 3. The PCA biplot case scores representation summarizes the possible relationship between the mycotoxins content in different regions during the two harvest years. It can be noted that the incidence and level of mycotoxins varied in samples from different wheat–growing regions. Between samples of the two years significant differences were recorded. The cumulative differences between 2010 and 2011 were well represented for wheat samples from both counties, Figure 2(a, b). The PCA data of the wheat samples harvested in 2010 year using the correlation matrix presented 47.26% variance for the first component (PC 1) and 29.40% variance for the second component (PC 2). The data points corresponding to the highest contents of mycotoxins were: Turnu (Arad County) for DON (3390 ppb), Munar (Arad County) for ZON (1000 ppb) and Tormac (Timis County) for FUMO (1180 ppb). Inside the ellipse we can appreciate the content of each sample according to the distance from the data points of sample to the analyzed mycotoxins data vectors, Figure 3a. The PCA applied to data corresponding to mycotoxins content registered in wheat samples harvested in 2011 using the correlation matrix led to a variance value of 58.51% for the first component (PC 1) and 29.02% for the second component (PC 2), so we chose to use the distribution only on the first two axes (with highest correlation variance), Figure 3b.

It was noticed that, the wheat samples from Semlac (Arad County) were characterized by the highest content of DON followed by Izvin (Timis County) (quarter I of the ellipse) while for wheat grain samples from Turnu (Arad County) it was registered a high level of OTA followed by Liebling (Timis County) and Simand (Arad County) (quarter II of the ellipse).

The localities distributed in quarter III were represented for ZON level. Thus, the highest content of ZON was registered in wheat grain samples from Calacea, Pesac (Timis County), Pecica, Peregu Mic (Arad County), etc. The highest content of FUMO was found in wheat samples from Curtici (Arad County) and Izvin (Timis County) (quarter IV of the ellipse).

### Histopathological evaluation

**Figure 4:**
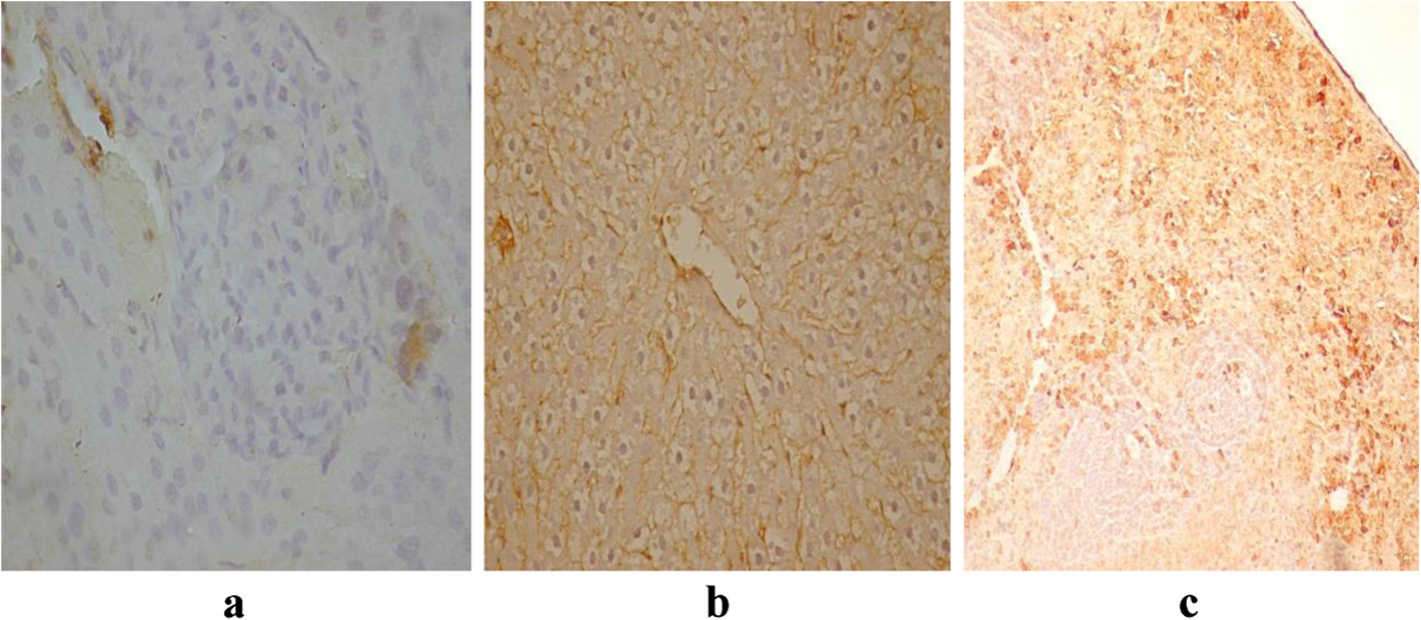
DON toxicity (VEGF: a: kidney; b: liver; c: spleen).

**Figure 5:**
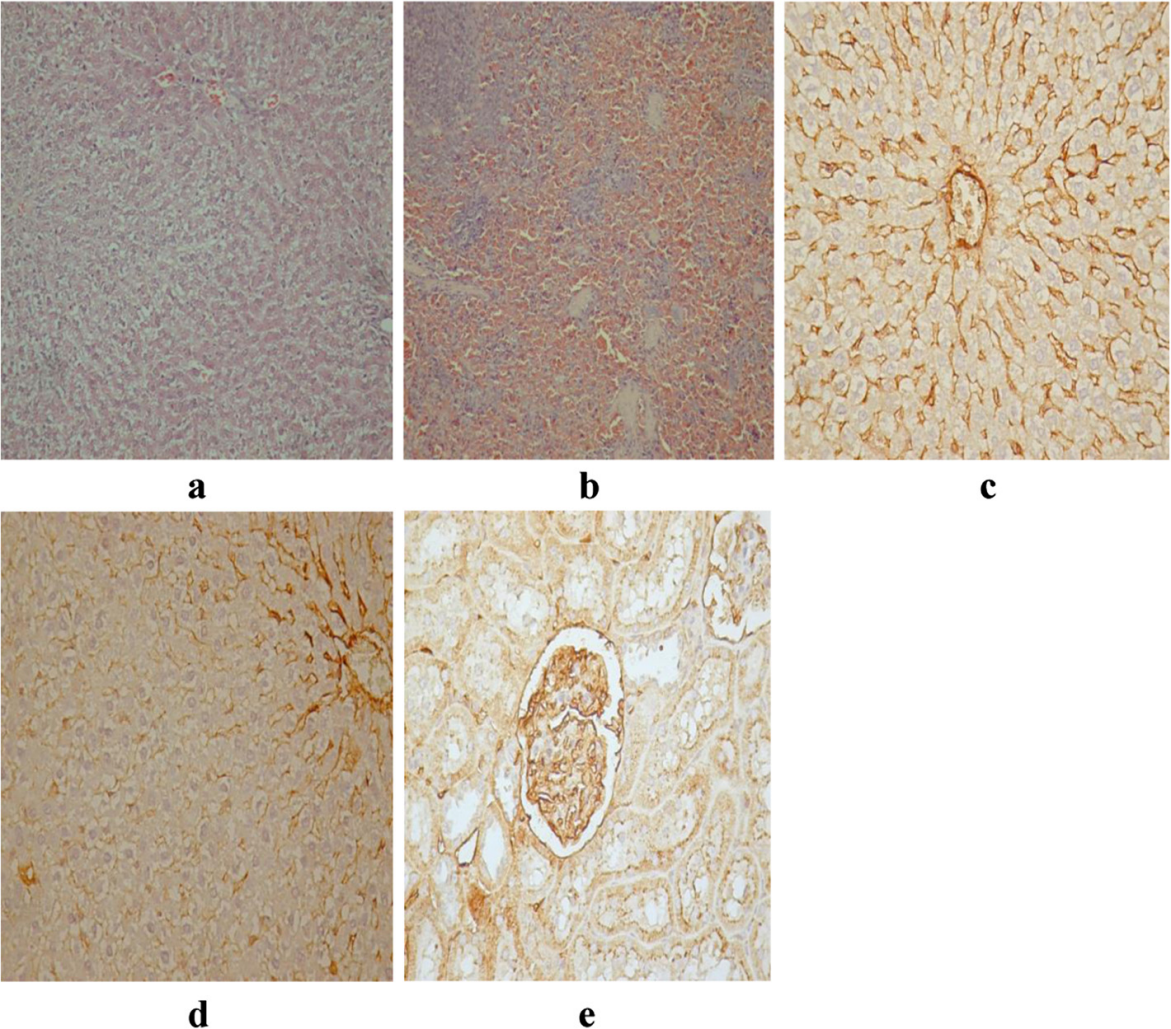
OTA toxicity (a: HE liver; b: HE spleen; c: VEGF liver normal; d: VEGF liver toxicity; e: VEGF kidney).

**Figure 6:**
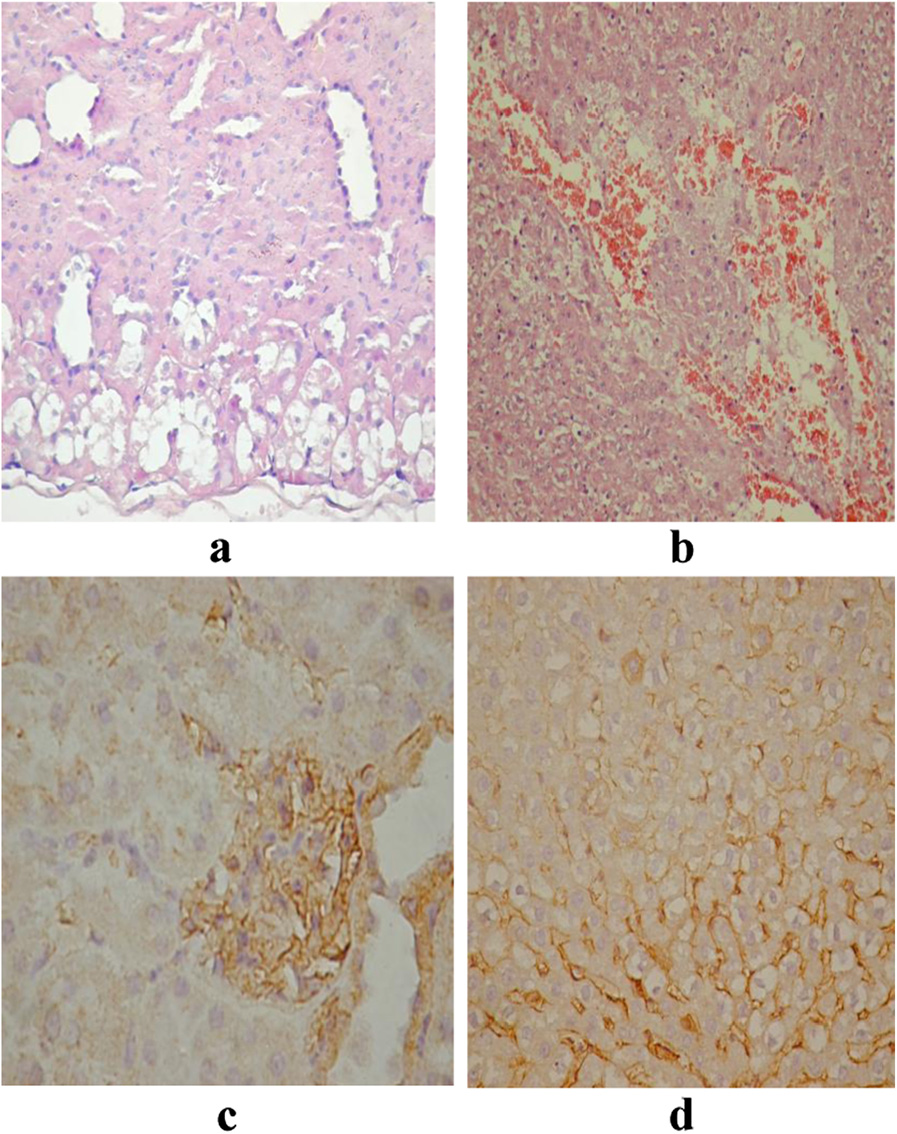
Fumonisin toxicity (a: HE kidney; b: HE liver; c: VEGF kidney; d: VEGF liver).

**Figure 7:**
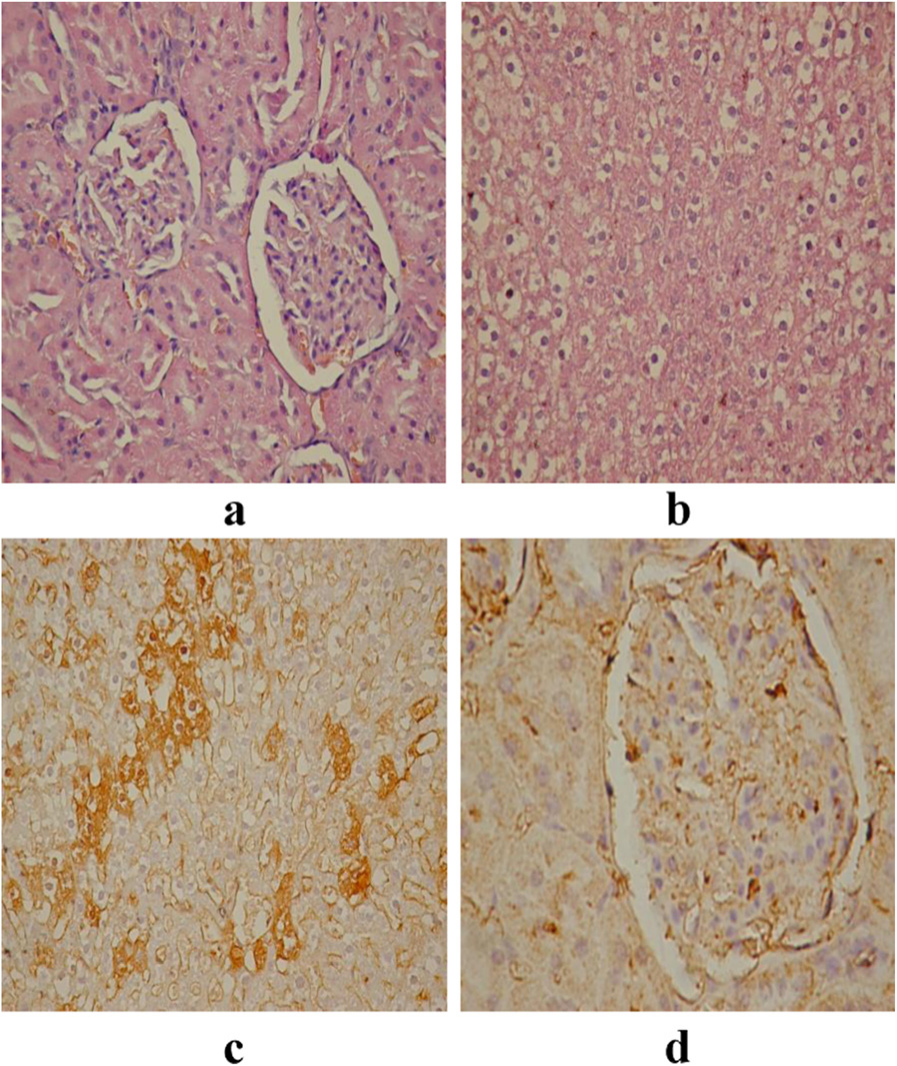
**ZON toxicity (a: HE kidney; b: HE liver; c**: **VEGF liver; d: VEGF kidney).**

The toxic effect of mycotoxins consumption expressed as histopatological evaluation is presented in Figures 4, 5, 6 and 7. Histopathological effects regarding the kidney and heart lesions after acute exposure of laboratory animals to lethal DON doses were reported in the previous researches [44]. In our study, after one month of feeding with wheat grain natural contaminated with DON, the histopathological and immunohistochemical evaluation performed by HE staining technique as well as by assessing of VEGF revealed intense destruction of cells kidneys from the deep cortical area with changes on kidney’s medulla, renal corpuscles fibrosis and lack of capillaries. These alterations indicate loss of functionality, intense destruction of liver parenchyma and disorganization of portal spaces, as a result of intense toxicity. In kidneys VEGF is absent in all analyzed areas, Figure 4a. VEGF expression in liver was absent on damaged area and present in the hepatocytes surrounding the central lobular vein, Figure 4b. In spleen it was observed a high expression of VEGF caused by the elevated number of inflammatory cells, Figure 4c.

OTA is a potent nephrotoxin, thus, repeated administration of OTA to rats resulted in high incidences of renal tumors that arising from the proximal tubular epithelial cells [45,46]. The results reported by Abdu *et al*., 2011 [47] revealed significant histological lesions in the case of mice treated with OTA. The lesions included global congestion in the renal tissue and loss of demarcation between the cortex and medulla. In terms of toxicity induced by OTA, our results highlighted about 30% peripheral necrosis in liver with maintaining of zonal hepatocytes and the presence of mixed normal and necrotic areas, Figure 5a. Also, 10% from renal corpuscles are affected, as can be seen from Figure 5b. The presence of VEGF expression in liver hepatocytes around sinusoid capillaries can be seen in Figure 5c and a reduced of VEGF expression can be observed near necrotic areas, Figure 5d. However, kidneys have intense and normal VEGF expression, Figure 5e.

Previous studies performed on mice, rats and rabbits revealed that the liver and kidneys were the major targets of FUMO toxicity, the dominant morphologic change consisting in the individual cell necrosis [48]. The histopathological effects as a result of FUMO toxicity are shown in Figure 6. The kidneys shown a massive damage affecting renal cortex, fibrosis and maintaining of renal corpuscles, having a reduced filtration space and low fibrosis, Figure 6a. Liver was characterized by the presence of a massive hepatocytes necrosis on a large area that suggests an intense toxicity, dilatation of important vessels, sinusoid capillaries from liver parenchyma and portal veins, and reducing the liver parenchyma, Figure 6b. Similar results, regarding the toxicological effect of FUMO, were reported by Domijan *et al*., 2008 [49]. In kidneys was recorded a high expression on medulla tubular structure and reduced or absent on cortical area or on kidneys corpuscles, Figure 6c. Bucci *et al*., 1998 [48] reported the degeneration of renal tubular epithelium at doses in the range 99–484 ppm in males as well as a minimal to mild hepatotoxicity. VEGF expression in liver was present on sinusoid capillaries and absent on necrotic area, Figure 6d.

With respect to ZON toxicity the kidneys presents a normal expression, Figure 7a. Thus, the liver shown without capillary dilatation, the portal areas are missing and hepatic parenchyma is maintaining, Figure 7b. VEGF evaluation indicated in kidneys moderate expression in corpuscles and renal tubes, Figure 7d, and in liver a mixed expression correlated with mixed normal and necrotic areas. The ratio of damaged/normal area is about 50%, Figure 7c.

## Experimental research

### Wheat samples

A total number of 52 winter wheat samples destined for animal feed were randomly collected from individual farmers from the west part of Romania The samples were collected from the harvest of two consecutive years (2010 and 2011, 26 of each year) from two regions with tradition in cereals cultivation (Arad and Timis counties). These regions cover 13 rural localities, respectively Curtici, Munar, Pecica, Peregu Mic, Semlac, Socodor, Simand, Turnu (Arad County) and Calacea, Izvin, Liebling, Pesac, Tormac (Timis County). From each locality were taken two wheat grain samples. Due to the irregular mycotoxins distribution a proper sampling was ensured according the EU requirements [35]*.* Samples were stored at 4°C until the analysis.

### Mycotoxins analysis

The method used in this study was enzyme-linked immunosorbent assay (ELISA) [50]*.* Commercial ELISA kits for mycotoxins identification were purchased from R-Biopharm: FAST OCHRATOXIN A R5402, ZEARALENON SC, R 5502, DON R5901, FUMONISIN R5602.

### Method validation

Prior to analysis of the samples, the ELISA method was validated to ensure data quality. The method validation was carried on reference certificated materials produced by R-Biofarm. Validation of this method was carried out by determination of recoveries, the standard deviations (SD), the repeatability (RSDr) and reproducibility (RSDR), the minimum Limit of Detection (LOD) and Limit of Quantification (LOQ). The summary of validation data of ELISA method was presented in the Table 3. These performance characteristics were in agreement with the limits accepted by the Commission’s Regulation for official methods of mycotoxins analysis [42].

**Table 3:** Summary of validation data for ELISA method

**Validation data**	**OTA**	**ZON**	**DON**	**FUMO**
LOD (ppb)	2.214	25.69	110.0	232.00
LOQ (ppb)	4.038	44.60	220.0	462.36
Average recoveries ± SD (%)	98.83 ± 9.4187	80.55 ± 6.0545	75.93 ± 0.2400	89.52 ± 0.2048
Repeatability (RSDr) (%)	13.9470	1.0680	0.0397	0.1982
Reproducibility (RSDR) (%)	10.16	0.887	0.107	0.209

### Sample analysis

Sample preparation and test method were conducted according to the instructions outlined in the R-Biopharm kits ELISA, as is described below. All samples were grounded to a fine powder (over 75% of the material passed through a 20-mesh sieve). The ground samples (5 g) were extracted with 25 ml mixture methanol:water 70:30 (v/v) for ZON and FUMO analysis, 12.5 ml of this mixture for OTA determination, respectively 100 ml distilled water for DON analysis, then shaken in a Warring blender at high speed for 20 min and filtered through a Whatman (Maidstone, UK) filter paper (No. 1). One ml filtrate was diluted at 1:1 with distilled water, for OTA and ZON, 1:13 for FUMO. The filtered extract was used directly for mycotoxins analysis. Standard solutions and prepared samples (50 μl) were mixed with 50 μl of enzyme conjugate in individual dilution wells. Antibody solution (50 μl) was added and mixed gently by shaking the plate manually and incubated for 10 min. at room temperature. Wells were washed three times with 250 μl distilled water. Substrate (100 μl) was added to each well and incubated for 5 min at room temperature. Following the addition of stop solution (100 μl) to each well, the intensity of the resulting yellow color was measured at a wavelength of 450 nm using an ELISA 96-well plate reader (PR-1100, Bio-Rad Laboratories, USA). The log-logit sheets supplied with the kits were used to generate a standard curve and to calculate the mycotoxins content in the samples. All determinations were performed in triplicate.

### Statistical analysis

The statistical evaluation of the experimental data was made using MVSP 3.1 and PAST 2.14. Principal Component Analysis (PCA) is a method that projects a multivariate dataset to a new coordinate system by determining the eigenvectors and eigenvalues of a matrix, facilitating visualization of the data. It is a procedure which uses an orthogonal transformation to convert a set of observations of possibly correlated variables into a set of values of uncorrelated linearly variables named principal components [51,52]. PCA model is used to identify patterns in data, and to highlight the data similarities and differences. A principal component for a given set of n-dimensional data, described by equation (1), is a linear combination of the original variables with coefficients equal to the components of an eigenvector of the correlation or covariance matrix [53]. Principal components are usually sorted by descending order of the eigenvalues - i.e. the first principal component corresponds to the eigenvector with the maximal eigenvalue [51,52].

(1) $$PC_{j}=\sum_{i=i}^{n}ai_{j}x_{i}$$

Where: PC – principal components; n – number of variables; x_i_ – the variables; a_ij_ – loading of the variable x_i_ on the principal component j.

### Toxicological and histopathological studies

Animal studies were conducted on Sprague Dawley male rats, 2 month old, purchased from Charles River (Sulzfeld, Germany). The work protocol followed all NIAH-National Institute of Animal Health rules: animals were maintained during the experiment in standard conditions: 12 h light–dark cycle, feed and water ad libitum, temperature 24°C, humidity above 55%. All experiments were approved by Bioethical Committee of Victor Babes University of Medicine and Pharmacy, Timisoara. The animals were divided into five experimental groups (E1-DON group, E2-TA group, E3-FUMO group, E4-ZON group) each of five animals and placed into cages. Rats were fed diets containing 100% naturally moulded wheat according to daily feed consumption/rat. The control group E5 received non-contaminated wheat. The experiment lasted 30 days with a daily check of the animals’ health state. After one month, histopathological changes were monitored in the liver, kidney and spleen of treated and control animals.

The histopathological and immunohistochemical evaluation was performed by HE staining technique and then by assessing the VEGF in studied samples. Biopsies were paraffin embedded and five micrometers sections were performed from each paraffin block. On *hematoxylin and eosin* stained sections we evaluated the morphology of liver, kidney, heart and spleen and we selected slides for VEGF immunostaining. Dewaxed and rehydrated slides were subjected to antigen retrieval step for 20 minutes at 99°C using a high pH antigen retrieval solution. After hydrogen peroxide inhibition step, specimens were incubated with anti VEGF antibodies (clone VG1, 1:25, Dako, Carpinteria, USA) followed by visualization with ADVANCE–HRP system and 3, 3′diaminobenzidine as chromogen. The counterstain was carried out using modified Lillie’s hematoxylin. Microscopic evaluation of specimens was done by using *Nikon Eclipse E600 Microscope* and images were captured with Canon camera attached to the microscope and processed with *LUCIA G image analyzer*.

## Conclusions

This study reported the mycotoxins incidence in wheat harvested in Western Romania during two harvest years. It was determined that DON, ZON and FUMO occur as a result of infection of the *living plants* by the relevant Fusarium *species,* but mycotoxins also occurred during storage – OTA, produced by *Aspergillus* and *Penicillium.* Our results highlight that for wheat samples harvested from Western Romania a high incidence of mycotoxins produced by *Fusarium* species have been recorded (DON and ZON). Nevertheless, the wheat samples are suitable for animal nutrition, none of the samples exceeding the stipulated maximum limits for cereals used as feed. The incidence of mycotoxins in cereals was influenced by seasonal weather conditions. Romania is located in the continental climate with optimal conditions for fungal development. Results indicate that PCA may be successfully applied as rapid method for localities discrimination depending on the content of mycotoxins registered in wheat grain samples. Considering all these factors, it can be concluded that measures to control mycotoxins content in cereals are necessary and that the development of strategies focused on contamination reduction in the affected areas is also mandatory. Regarding the histopathological assessment our results highlighted that the most toxic compounds after a short time feeding with natural contaminated wheat were FUM and DON. They produced significant tissue lesions in liver and kidney of rats and reduced or determined the absence of VEGF expression which indicates no possibility for recovery on these areas.

## Abbreviations

FUMO: Fumonisin; DON: Deoxynivalenol; OTA: Ochratoxin A; ZON: Zearalenone; HE: Hematoxylin; VEGF: Vascular endothelial growth factor; ELISA: Enzyme-linked immunosorbent assay; SD: Standard deviations; RSDr: Repeatability; RSDR: Reproducibility; LOD: The minimum Limit of Detection; LOQ: Limit of Quantification; MAL: Maximum admitted level; HE: Hematoxylin-eosin; VEGF: Vascular endothelial growth factor.x`

## Competing interests

The authors declare that they have no competing interests.

## Authors’ contributions

EA and IR designed the study and coordinated the preparation of the manuscript, carried out the mycotoxins analysis by ELISA test; MAP, GP and CT co–worked on the sample collection, performed the mycotoxins analysis and helped to draft the manuscript, CAD and AMC performed the toxicological and histopathological experiment, DMB carried out the statically study. All authors read and approved the final manuscript.
